# The Impact of Fecal Microbiota Transplantation on Gastrointestinal and Behavioral Symptoms in Children and Adolescents with Autism Spectrum Disorder: A Systematic Review

**DOI:** 10.3390/nu17132250

**Published:** 2025-07-07

**Authors:** Anna Liber, Małgorzata Więch

**Affiliations:** Department of Nutrition, Institute of Mother and Child, Kasprzaka 17a, 01-211 Warsaw, Poland

**Keywords:** autism, fecal microbiota transplantation, gastrointestinal symptoms

## Abstract

**Background**: Gastrointestinal (GI) symptoms, often reported by individuals with autism spectrum disorders (ASD), may impair functionality and exacerbate behavioral symptoms. Gut dysbiosis has been identified as a potential environmental factor influencing these symptoms through gut-brain axis dysregulation. Fecal microbiota transplantation (FMT) is a promising therapeutic strategy with potential to alleviate symptoms. This review systematically evaluates the efficacy and safety of FMT in GI and ASD-related symptoms. **Methods**: This systematic review followed PRISMA 2020 guidelines and was registered in PROSPERO. The review included clinical trials on FMT in children and adolescents with ASD, published up to October 2024. The bias assessments were performed using Cochrane tools. Outcomes focused on changes in GI and ASD-related symptoms using scales selected by the authors. **Results**: This systematic review included two RCTs and seven before-and-after studies. Improvements in GI and ASD-related outcomes were reported in all before-and-after studies, whereas the results of RCTs were inconsistent. The before-and-after studies showed a high risk of bias, while the RCTs demonstrated a low risk. **Conclusions**: Although many studies have been conducted, the methodological limitations of some and contradictory findings of others make it difficult to draw clear conclusions about the effectiveness of FMT in children with ASD. Variations in intervention protocols underscore the importance of establishing standardized FMT procedures in future rigorously designed trials.

## 1. Introduction

Autism spectrum disorder (ASD) is a group of neurodevelopmental conditions characterized by persistent deficits in social communication and interaction, as well as restrictive and repetitive patterns of behavior. Currently, no reliable biomarker for ASD has been identified; thus, diagnosis relies on information provided by parents or caregivers regarding the child’s activity and development, combined with specialists’ observations of the child’s behavior [1].

The prevalence of ASD has increased significantly worldwide in recent decades, ranging from 0.4% to 1.7%, depending on the continent and country where the analyses were performed [2]. This rise represents a significant public health concern highlighting the need for the development and evaluation of novel therapeutic approaches.

Although the etiology of ASD is not yet fully understood, both genetic and environmental factors are believed to play a role in its development [3]. Among environmental factors, gut microbiota has emerged as a potential agent influencing ASD symptomatology [4].

The composition of gut microbiota in children with ASD differs from that of neurotypical individuals. Numerous studies have reported a higher abundance of the *Firmicutes* phylum, particularly of potentially harmful spore-forming *Clostridium*, alongside a reduced abundance of potentially beneficial *Bifidobacterium* [5].

Dysbiosis, an imbalance in the diversity, composition, and metabolic activity of the gut microbiota, is also prevalent in individuals with functional bowel disorders, resulting in gastrointestinal (GI) symptoms such as diarrhea, constipation, abdominal pain, or bloating [6]. Moreover, dysbiosis has been observed in various neurological conditions, including autoimmune diseases such as multiple sclerosis, as well as neurodegenerative disorders like Parkinson’s disease and Alzheimer’s disease, suggesting its broader relevance beyond the GI tract. Notably, these neurological conditions are themselves frequently accompanied by GI symptoms, including altered bowel habits [7].

The prevalence of GI problems is significantly higher in children with ASD than in their neurotypical peers, with estimates reaching up to 69% [8]. These symptoms are associated with functional disorders of the GI tract, potentially linked to gut-brain axis dysregulation. While studies have found correlations between GI problems and the severity of ASD-related symptoms, a causal relationship has not yet been established [9].

Interventions targeting gut microbiota present a promising therapeutic avenue for children with ASD, offering the potential not only to alleviate frequent GI symptoms but also to reduce the severity of behavioral deficits.

Fecal microbiota transplantation (FMT) involves the transfer of fecal material from a healthy donor into the intestine of a recipient to treat conditions associated with gut microbiota alterations. This method has been well-established as an effective treatment for recurrent *Clostridioides difficile* infections, achieving up to 90% success rates [10]. The impact of FMT on ASD-related symptoms has also been investigated in animal models to elucidate potential mechanisms and therapeutic effects. Abuaish et al. examined FMT in Fmr1 knockout mice, a model of Fragile X syndrome—a heritable neurodevelopmental disorder characterized by autistic-like behaviors, including social deficits and cognitive impairments [11]. The study showed that FMT improved social and cognitive functions, restored gut microbiota composition—particularly by increasing *Akkermansia muciniphila*—and reduced neuroinflammation by normalizing TNFα levels and microglial activation. Similarly, Zheng et al., demonstrated that human-derived FMT alleviated behavioral abnormalities in BTBR mice, another ASD model, and linked these improvements to altered vitamin B6 metabolism involving specific *Bacteroides* species. These findings highlight the therapeutic potential of FMT in alleviating ASD-related symptoms through microbiota-driven modulation of host metabolic pathways [12]. FMT has also been tested in patients with ASD, showing potential benefits in addressing associated symptoms. Two systematic reviews assessing the effects of FMT in children with ASD were published in 2023, including studies published up to 2022 [13,14]. Both concluded that FMT holds promise in alleviating GI and behavioral symptoms. However, as new studies have been published since these reviews, we decided to update the current knowledge on this topic. Therefore, we conducted a systematic evaluation of the efficacy and safety of FMT on gastrointestinal and behavioral symptoms in individuals with ASD.

## 2. Methods

We conducted this systematic review using the Preferred Reporting Items for Systematic Reviews and Meta-Analyses (PRISMA) 2020 guidelines [15]. The study protocol was registered in the PROSPERO database under the identifier CRD42024569359.

### 2.1. Eligibility Criteria

Studies were eligible for inclusion in the systematic review if they met predefined criteria. These included all types of clinical research designs, such as randomized controlled trials (RCTs), non-randomized interventions (e.g., before-and-after studies), as well as observational and cohort studies, provided they were published in English up to October 2024. The population of interest consisted of individuals with ASD, diagnosed according to the criteria specified by the study authors. The intervention under consideration was FMT, delivered through any route, with outcomes focused on gastrointestinal and ASD-related symptoms assessed using validated rating scales. Case reports and case series were excluded from the review. Although RCTs are the preferred study design for evaluating intervention effects due to their low risk of bias, non-randomized studies were also included. This decision was based on the likelihood of a limited number of available RCTs.

### 2.2. Information Sources

Two reviewers independently searched three databases, MEDLINE via PubMed, EMBASE, and CENTRAL (Cochrane Library), for trials meeting the inclusion criteria. Additionally, they examined the reference lists of included studies and relevant systematic reviews.

### 2.3. Search Strategy

The search strategy was developed using free-text terms and standardized vocabulary, including Medical Subject Headings (MeSH) for MEDLINE and CENTRAL and Emtree terms for EMBASE. Two groups of search terms were applied. The first group targeted the diagnosis of participants, including terms such as *autism*, *autism spectrum disorder*, and *Asperger syndrome*. The second group focused on the intervention, using combinations of terms describing FMT, such as *fecal*/*fecal*/*microbiota*/*bacteria* paired with *transfer*/*transplant*/*transplantation*/*therapy*. Detailed search strategies for each database are provided in Supplementary Table S1.

### 2.4. Selection and Data Collection Processes

The results from the database searches were independently screened by reviewing the titles and abstracts of each record. Full texts of potentially relevant articles were subsequently analyzed to determine their final eligibility. Any disagreements were resolved through discussion to reach a consensus. Data from eligible studies were extracted using a standardized data extraction form by one reviewer (AL) and verified by a second reviewer (MS). The collected information included the author, publication year, study setting, sample size, age of participants, ASD diagnostic criteria, detailed information about the intervention, types of outcomes related to GI and ASD symptoms, and methodological quality.

### 2.5. Study Risk of Bias Assessment

The risk of bias for included studies was evaluated using the Cochrane Collaboration tools: ROBINS-I tool for non-randomized studies and the RoB 2 tool for randomized controlled trials. Both tools assess multiple domains of bias. For non-randomized studies (ROBINS-I), seven domains were assessed: confounding, participant selection, intervention classification, deviations from intended interventions, missing data, outcome measurement, and selective reporting. Each domain was rated as low, moderate, serious, or critical risk of bias. For randomized controlled trials (RoB 2), five domains were analyzed: the randomization process, deviations from intended interventions, missing data, outcome measurement, and selective reporting. The results were categorized as low risk, some concerns, or high risk of bias.

The overall risk of bias for each study was determined based on the highest risk rating across the domains. The assessment was performed independently by both reviewers. Any disagreements were resolved through discussion.

### 2.6. Effect Measures

The primary outcomes analyzed were changes in GI and ASD-related symptoms before and after the intervention. In RCTs, mean difference (MD) with a 95% confidence interval (CI) was applied for continuous outcomes to compare the experimental and control groups. When a placebo group was not available, the analysis focused on the changes observed within each study arm from baseline to the end of the study. The secondary outcomes encompassed the adverse effects documented in the analyzed studies.

Wang et al. [16] presented the trial results as within-group changes during the study period in both the experimental and placebo groups. We contacted the authors to request the raw data in order to calculate the MD and 95% CI between the experimental and placebo groups.

## 3. Results

### 3.1. Study Selection

**Figure 1:**
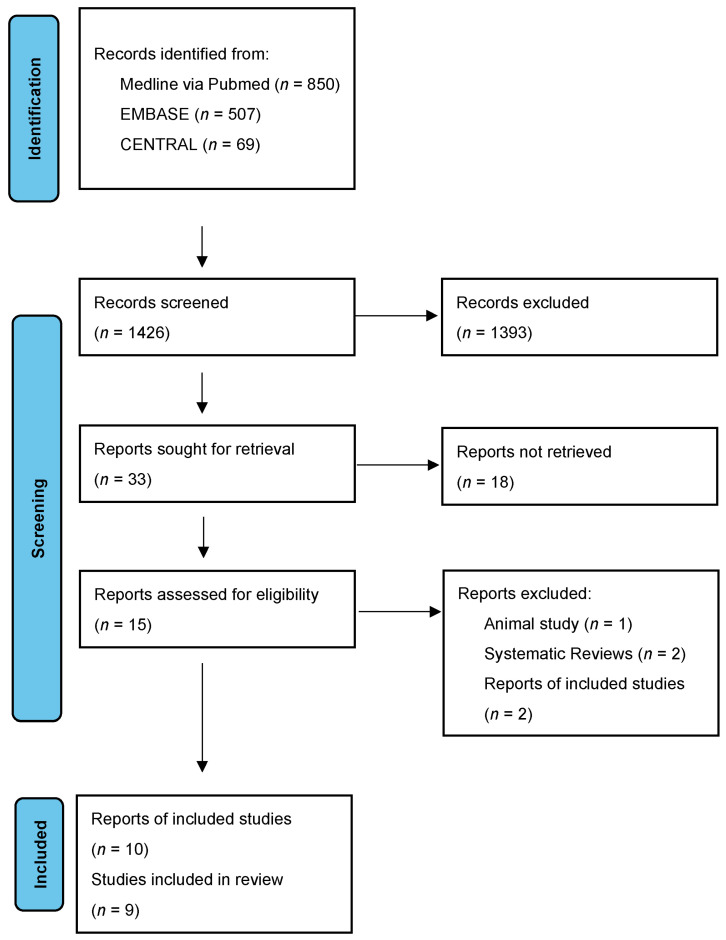
Flow diagram of the study selection process.

The study selection process followed the PRISMA guidelines and is illustrated in a flow diagram (Figure 1).

Two systematic reviews on the same topic, both published in 2023, were identified [13,14]; collectively, they included four studies published up to 2022. In the present review, we identified five additional studies published in 2023 and 2024.

### 3.2. Study Characteristics

The review included two RCTs [16,17] and seven before-and-after studies. Data in two before-and-after studies were collected retrospectively [18,19], while in five studies [20,21,22,23,24], they were collected prospectively. For the study by Kang et al., we identified two reports: one presenting results after the intervention [21] and the other with a two-year follow-up post-treatment [25]. The RCTs were double-blinded and placebo-controlled, whereas the before-and-after studies lacked control groups. The included studies differed in sample sizes and intervention protocols.

ASD-related outcomes were assessed using tools such as the Aberrant Behavior Checklist (ABC), the Childhood Autism Rating Scale (CARS), the Social Responsiveness Scale (SRS), and the Vineland Adaptive Behavior Scales (VABS). GI outcomes were measured using the Gastrointestinal Symptom Rating Scale (GSRS) and the Bristol Stool Form Scale (BSFS).

The total sample size across all studies was 394, with individual study sizes ranging from 18 to 113 participants. The age of participants spanned from 2 to 17 years. None of the included studies stratified data by age or sex, nor did they consider the potential influence of hormonal changes—such as those occurring during puberty—on gut microbiota composition or behavioral outcomes. Moreover, the majority of participants in the included studies were boys, which reflects the higher prevalence of autism spectrum disorder in males. All studies examined FMT as the primary intervention, employing various delivery methods, including oral capsules, nasojejunal tubes, colonoscopic administration, and transendoscopic enteral routes. The duration of interventions varied, ranging from two 1-day courses per week to four 6-day courses per month.

The characteristics of included studies are presented in Table 1.

To date, no standardized intervention model for FMT in children with ASD has been established. The intervention protocols in the included studies varied significantly; therefore, we decided to present the detailed methods used in Table 2. Insight into the applied intervention methods may assist in planning future studies.

### 3.3. Results of Interventions

The summary of findings is presented in Table 3.

### 3.4. Gastrointestinal Symptoms

Six studies [18,19,20,21,23,24] evaluated gastrointestinal symptoms using the Bristol Stool Form Scale (BSFS), which classifies stool into seven types based on shape and consistency. All studies reported some improvements. Kang et al. [21] observed a significant reduction in the number of days with abnormal stools (types 1, 2, 6, or 7) following the intervention and during follow-up. Similarly, N. Li et al. [23] found a marked decrease in the frequency of days with both soft (types 6 or 7) and hard stools (types 1 or 2). Pan et al. [18] showed an increase in the number of children with normal stool consistency after the second to fifth FMT courses, as well as a reduction in the number experiencing constipation after each set of five courses; however, no improvement was observed following the first course. Chen et al. [20] and Liu et al. [24] documented overall improvements in BSFS scores post-treatment, whereas Y. Zhang et al. [19] observed such improvement only among children with constipation.

The authors of five studies [16,20,21,22,23] employed the Gastrointestinal Symptom Rating Scale (GSRS) to evaluate improvements in GI symptoms. The GSRS, a self-reported, seven-point Likert scale originally validated in adults, measures the severity of GI symptoms across domains such as abdominal pain, reflux, indigestion, diarrhea, and constipation [26]. In the studies involving children with ASD, symptom assessments were conducted by parents. Notably, this scale has not been validated for use in children with ASD or for parent-reported evaluations.

All studies reported significant reductions in GSRS scores. In the before-and-after studies, reductions ranged from 35% to 82% from baseline to post-treatment, and from 32% to 77% during the follow-up phase. In the RCT conducted by Wang et al. [16], a statistically significant difference was observed between the experimental and placebo groups (MD = −10.07, 95% CI: −13.68 to −6.46).

### 3.5. ASD-Related Symptoms

All included studies evaluated the effects of the intervention using the ABC scale, a parent- or caregiver-reported measure designed to assess behavioral symptoms in individuals with developmental disorders. Significant improvements in ABC scores following FMT courses were reported in six before-and-after studies [18,20,21,22,23,24]. In the study by Y. Zhang et al. [19], a significant improvement after the second FMT course was observed only in children with constipation, while no changes were noted after the first course or in children without constipation. In contrast, the findings of the two RCTs [16,17] revealed no significant reduction in ABC scores between the experimental and placebo groups. However, in the study by Wang et al. [16], significant improvements after the intervention, compared to baseline, were observed in both groups when analyzed separately.

Seven studies used the CARS—15-item rating scales completed by the clinician to assess the severity of ASD symptoms, based on direct observations and information provided by caregivers or parents, to evaluate behavioral symptoms. Five before-and-after studies [18,20,21,22,23] reported significant improvements following the intervention. In the study by Y. Zhang et al. [19], a significant improvement was observed only after the second FMT course in children with constipation, whereas children without constipation improved after both the first and second courses. Similarly, in the RCT conducted by Wang et al. [16], a significant difference in changes in CARS scores was observed between the experimental and control groups (MD = −2.12; 95% CI: −3.82 to −0.41).

The authors of five studies incorporated the SRS, i.e., a standardized questionnaire designed to assess social communication difficulties, restricted interests, and repetitive behaviors, typically completed by parents or caregivers. Significant improvements following the intervention were reported in three before-and-after studies [21,22,23]. In the study by Kang et al. [21], this positive effect persisted during follow-up periods of 8 weeks and 2 years, while Y. Li et al. [22] observed sustained results at 8 weeks post-treatment. In contrast, N. Li et al. [23] reported that the improvement was reversed at 8 and 12 weeks after the end of the intervention. Wang et al. [16] also observed significant improvement in FMT compared to the placebo group (MD 12.91 95% CI −21.40–−4.42). In contrast, a similar effect was not observed in the study by Wan, where SRS scores were comparable between the experimental and placebo groups.

Kang et al. [21] additionally used the Vineland Adaptive Behavior Scales (VABS) which assess adaptive behaviors in four domains: communication, daily living skills, socialization, and motor skills. The authors converted raw scores into age-equivalent scores and observed that the average developmental age increased by 1.4 years within 8 weeks after treatment. However, the developmental age remained lower than the participants’ chronological age. During the subsequent two years of follow-up, the VABS age-equivalent scores continued to improve, resulting in a total increase of 2.5 years.

The same scale was also utilized in the RCT by Wan et al. [17]. While the overall alteration in the VABS score was similar across groups, the authors identified a significant difference in the socialization domain (MD 5.05, 95% CI 0.31–9.79) and the play and leisure subdomain during the follow-up period (MD 1.36, 95% CI 0.19–2.53).

### 3.6. Adverse Events

Six studies [17,19,21,22,23,24] investigated the safety and tolerability of FMT. Among these, four studies [17,21,22,23] reported adverse effects (AEs), none of which were classified as serious. Most AEs were transient. The most commonly reported symptoms included rash, fever, irritability, hyperactivity, insomnia, depression, restlessness, lymph node pain, urinary frequency, diarrhea, vomiting, and nausea.

In contrast, two studies [19,24] reported no adverse outcomes, while three studies [16,18,20] did not address this aspect. Notably, in the RCT by Wan et al. [17], the incidence of AEs was comparable between the FMT and placebo groups, with 12 (23.1%) and 7 (13.7%) patients, respectively, experiencing one or more events.

### 3.7. Risk of Bias Assessment

**Figure 2:**
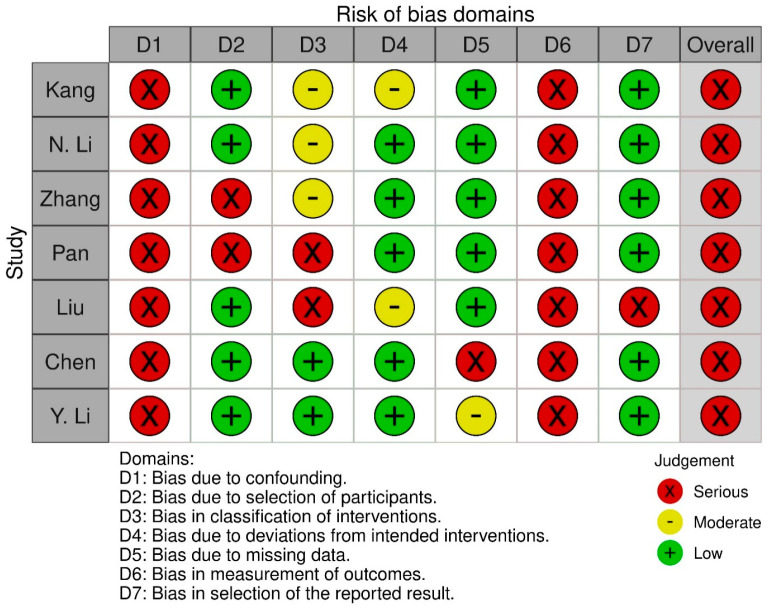
Risk of bias assessment for before-and-after studies using ROBINS-I tool [18,19,20,21,22,23,24].

The risk of bias assessment for before-and-after studies was performed with the use of the ROBINS-I and is presented in Figure 2. All before-and-after studies presented a serious risk of bias especially due to the lack of a control group.

**Figure 3:**
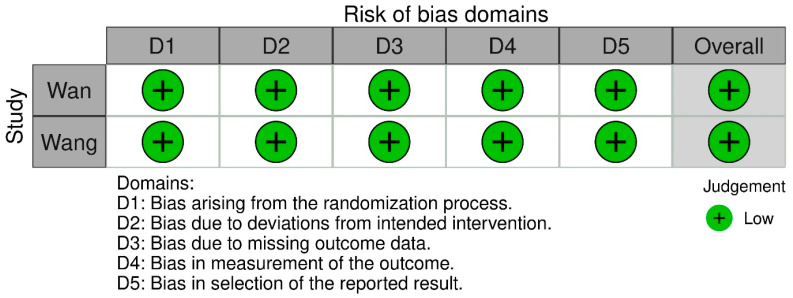
Risk of bias assessment for RCTs using the ROB 2 Tool [16,17].

The risk of bias in the RCTs was evaluated using the ROB 2 tool, with the results detailed in Figure 3. Both RCTs were determined to have a low risk of bias. However, the study by Wan performed a per-protocol analysis rather than an intention-to-treat analysis.

## 4. Discussion

GI symptoms are among of the most common comorbidities in children with ASD and have the potential to exacerbate behavioral symptoms and worsen a child’s functioning [9]. This systematic review highlights the potential of FMT for managing both GI and ASD symptoms.

Although numerous studies have investigated this issue, drawing definitive conclusions about the effectiveness of the intervention remains challenging due to methodological limitations in most studies and inconsistent findings among those with a low risk of bias. The available data suggest that the intervention is generally safe, with no serious adverse events reported and only mild, transient symptoms observed.

All seven included before-and-after studies demonstrated a marked improvement in GI and ASD-related symptoms following FMT; however, the findings should be interpreted with great caution due to the serious risk of bias caused by the lack of a control group and the potential impact of the placebo effect.

The observation period in all included studies was relatively short, with a maximum of 8 weeks post-intervention. Authors primarily focused on immediate post-interventional outcomes. However, Kang et al. [25] conducted a re-evaluation of participants two years after the conclusion of their study and found that improvements in both GI and ASD-related symptoms persisted, suggesting that the effect may be long-term. These sustained clinical benefits were accompanied by long-lasting changes in gut microbiota composition. Such findings suggest that FMT may exert durable therapeutic effects possibly related to successful engraftment of donor-derived microbial communities. In the study by Pan et al. [18], significant improvements in both gastrointestinal and behavioral symptoms were not observed after the initial FMT session but became apparent only after multiple administrations. This temporal pattern suggests a potential dose–response relationship, where cumulative exposure to transplanted microbiota may be necessary to achieve therapeutic benefit. Such findings underscore the importance of considering treatment duration and the number of FMT sessions when designing protocols. The observed improvement in gastrointestinal symptoms was clinically meaningful, with post-treatment response rates reaching up to 82% and 58% of participants maintaining symptom improvement at the two-year follow-up. Similarly, improvements in ASD-related symptoms were clinically relevant, with short-term reductions in ABC scores reaching up to 35% and sustained improvements of 24% observed at 2-year follow-up. In the CARS scale, symptom reductions ranged from 23% to as high as 47%, supporting the therapeutic impact of FMT and its potential for long-lasting effects.

Two randomized controlled trials (RCTs) reported conflicting outcomes, despite similarities in study populations and intervention protocols. In the study by Wan et al. [17], which had the largest sample size among the included trials (*n* = 113), no significant differences in ASD-related symptoms were found between the FMT and placebo groups. Both groups demonstrated post-treatment improvements, and the comparable incidence of adverse effects suggests the potential influence of placebo and nocebo effects. Conversely, a smaller study by Wang et al. [18] (*n* = 41) reported significant improvement in gastrointestinal symptoms. Improvements in behavioral symptoms were also observed on the CARS and SRS scales, although no significant changes were noted on the ABC scale.

Both RCTs assessed behavioral symptoms using the same scales, namely ABC and SRS. Although these are validated parent-reported questionnaires, they may be subjective and influenced by parental expectations, potentially contributing to the observed placebo effect. These tools are particularly susceptible to expectancy bias and may not reliably capture objective behavioral changes, especially in unblinded studies. In contrast, the CARS relies on interviews conducted by trained professionals, offering a more objective and reliable assessment. When planning future studies, it would be advisable to select diagnostic tools designed for use by professionals to ensure a more accurate assessment of therapeutic effects.

GSRS-based outcomes related to gastrointestinal symptoms should be interpreted with caution, especially for symptoms such as abdominal pain, hunger pain, nausea, and heartburn, as they relied on parental reporting. Notably, the scale may offer more objective assessments for stool-related symptoms, such as loose or hard stools. However, since the total GSRS score reflects a composite of all symptom domains, this may reduce its reliability and represent a limitation when applied in studies involving children with ASD.

The Bristol Stool Form Scale (BSFS) is a validated tool for assessing stool consistency and was applied in six before-and-after studies, though not in any RCTs. Recommended by the Rome Foundation Pediatric Subcommittee for use in pediatric irritable bowel syndrome (IBS) trials alongside the Visual Analog Scale for abdominal pain [27], it enables reliable monitoring of stool consistency and frequency in children with ASD. The BSFS is limited to stool-related symptoms and does not assess the full spectrum of gastrointestinal complaints, such as abdominal pain, bloating, or reflux. As such, interpretation of GI symptom improvement based solely on BSFS scores is insufficient. There is a clear need for the development and validation of comprehensive tools tailored to children with communication difficulties that are capable of capturing a wider range of gastrointestinal symptoms.

The selection of appropriate outcome measures is not the only challenge faced by researchers planning similar studies. An equally important issue is the lack of a standardized protocol for conducting FMT in children with ASD. The FMT protocol may include preparatory procedures such as antibiotic priming, bowel cleansing, and the use of gastric acid suppressants for upper gastrointestinal delivery [28]. Among the included studies, only Kang et al. [21] applied all three components: 14 days of oral vancomycin, a 1-day liquid diet with bowel cleansing using polyethylene glycol, and omeprazole administered from day five before the procedure until the final day of treatment. In three studies [16,23,24], bowel cleansing was performed prior to FMT. In five studies, no preparatory procedures were undertaken. These differences raise the question of which components are essential to ensure the effectiveness of the intervention.

A key difference between the two included RCTs [16,17] was the use of preparatory procedures prior to FMT, which may have contributed to the differences in the results observed between the studies. In the study by Wang et al. [16], patients followed a three-day residue-free, semi-liquid diet and underwent bowel cleansing with polyethylene glycol. In contrast, Wan et al. [17] did not include any preparation. It is worth considering whether bowel cleansing prior to FMT contributed to the intervention’s effectiveness observed in the study by Wang et al. [16]. A metagenomic meta-analysis of 14 studies in adults with various conditions, though not with ASD, demonstrated that reducing microbial load through bowel lavage increases the likelihood of donor strain engraftment, a key determinant of FMT success [29]. Similarly, a French nationwide retrospective cohort study of patients with recurrent *Clostridioides difficile* infection found that inadequate bowel cleansing increased the risk of recurrence fivefold [30]. These findings suggest that incorporating bowel cleansing may improve FMT efficacy and should be considered in planning the FMT procedure.

Although antibiotic priming is a standard component of the FMT procedure for recurrent *Clostridioides difficile* infection, as it reduces the pathogenic bacterial load [31], its role in the management of chronic conditions remains unclear. Antibiotics are known to alter gut microbiota composition and have been implicated in the development of certain non-communicable diseases [32]. A prospective case-control study of patients with non-enteric infections found an association between antibiotic use and the onset of functional gastrointestinal disorders, particularly irritable bowel syndrome [33]. However, a cause-and-effect relationship has not been established. Considering the presented data, the use of antibiotics may be regarded as potentially harmful. Moreover, El-Salhy et al. [34] conducted an RCT in patients with IBS and reported a significant improvement in symptoms in the FMT group compared to the placebo group. Notably, this positive effect was achieved without the use of antibiotics. On the other hand, two metagenomic meta-analyses [29,35] revealed that antibiotic priming is associated with higher donor strain engraftment. However, these analyses were conducted in heterogeneous patient populations, primarily with recurrent *Clostridioides difficile* infection. The available data do not allow for a definitive conclusion on whether the use of antibiotics prior to FMT administration will enhance its effectiveness and impact symptoms in children with ASD.

In addition to antibiotic exposure, other factors known to modulate gut microbiota composition may influence the outcomes of FMT and should be systematically assessed and controlled for. To reduce potential confounding, six of the included studies excluded participants with recent use of antibiotics, probiotics, or immunosuppressants, applying washout periods ranging from one week to three months prior to FMT. For instance, Li N et al. [23] excluded the use of antibiotics and probiotics within seven days before screening; Kang et al. [21] excluded antibiotics within six months and probiotics within three months; Y Li et al. [22] excluded antibiotics, probiotics, and proton pump inhibitors within three months; Wang et al. [16] and Pan et al. [18] excluded antibiotics and probiotics within one month, with Wang et al. [16] additionally excluding immunosuppressants; and Liu et al. [24] excluded antibiotics within 24 h before FMT. However, other relevant dietary variables that may affect microbiota stability and engraftment potential—such as habitual diet, intake of prebiotics, fiber, or fermentable carbohydrates—were not controlled in included studies. To improve the accuracy and reproducibility of findings, future studies should evaluate and mitigate the impact of dietary factors affecting gut microbiota and FMT outcomes, including habitual diet, prebiotic intake, and fermentable substrates.

While procedural variables may influence the efficacy of FMT, emerging evidence indicates that its therapeutic potential is mediated by complex microbiota–host interactions along the gut–brain axis. This axis represents a bidirectional communication network linking the central nervous system and the gastrointestinal tract through neural, endocrine, immune, and metabolic pathways. Microbial metabolites—particularly short-chain fatty acids (SCFAs) such as butyrate, propionate, and acetate—play essential roles in maintaining gut barrier integrity, modulating systemic and neuroinflammatory responses, and regulating neurotransmitter synthesis. Disruptions in the gut microbiota can impair barrier function, leading to increased intestinal permeability and translocation of pro-inflammatory mediators, which may activate microglia and trigger neuroinflammation. Furthermore, gut microbes influence immune system maturation and can modulate neural cells via immune and hormonal signals. These interactions may disrupt neuroplasticity, synaptic signaling, and neurotransmission, ultimately contributing to behavioral alterations—core domains affected in ASD [36,37].

Seven of the included studies explored microbial composition, metabolite profiles, and gut-derived mechanisms, supporting the role of the gut–brain axis in mediating FMT’s therapeutic effects in ASD. Wan et al. [17] demonstrated that FMT significantly increased gut microbial alpha diversity and induced greater shifts in beta diversity compared to a placebo, despite having no effect on the core symptoms of ASD. However, in the subgroup of children with ≥20% donor strain colonization, greater improvements in adaptive functioning were observed, suggesting a potential association between donor engraftment and clinical benefit. Similarly, Liu et al. [24] reported increased alpha diversity and significant changes in beta diversity following FMT, including enrichment of *Prevotella_9* and a reduction in *Bacteroides*, *Flavonifractor*, and *Parasutterella*. These microbial shifts were associated with a decreased potential for the production of harmful metabolites (e.g., ammonia, p-cresol) and an enhanced capacity for SCFA synthesis. In the study by Kang et al. [21], children with ASD also showed a sustained increase in gut bacterial alpha diversity following FMT, with microbial profiles shifting toward those of neurotypical controls. This was accompanied by increased relative abundances of *Bifidobacterium*, *Prevotella*, and *Desulfovibrio*, as well as reduced UniFrac distances to donor samples, indicating partial engraftment. At the two-year follow-up, most participants maintained elevated bacterial diversity compared to baseline. Although the overall microbiota composition diverged from that of the donor over time, key beneficial taxa such as *Bifidobacterium* and *Prevotella* remained significantly enriched, suggesting lasting microbial changes associated with sustained improvements in gastrointestinal and behavioral symptoms. Y. Li et al. [22] reported increases in beneficial bacterial genera such as *Eubacterium_hallii*_group and *Anaerostipes*, alongside reductions in *Prevotella* and *Blautia*. The authors also analyzed the fungal component of the gut microbiota and observed shifts in composition, with an increase in *Aspergillaceae* and a decrease in *Saccharomycopsis*. Several bacterial and fungal taxa were found to correlate with core ASD symptoms, highlighting the potential relevance of both microbial domains in the therapeutic effects of FMT. Unlike previous studies, N. Li et al. [23] found no significant change in overall microbial diversity following FMT; however, microbial composition temporarily shifted toward that of typically developing controls. The treatment also led to changes in serum neurotransmitter levels, with decreased serotonin (5-HT) and GABA, and increased dopamine (DA), which correlated with improvement in GI symptoms. A lower baseline abundance of *Eubacterium coprostanoligenes* predicted a better clinical response, and its post-FMT reduction was associated with both GI improvement and lower GABA levels. This genus remained negatively correlated with serum GABA, suggesting a potential role in gut–brain regulation. Chen et al. [20] observed partial engraftment of donor-derived *Faecalibacterium*, *Prevotella*, and *Bifidobacterium* following FMT. Additionally, several metabolites—including biliverdin, acetylcarnitine, deoxycholic acid, and falcarindiol—were identified as potential mediators linking microbial changes to neurobehavioral outcomes, while *Collinsella* species were consistently negatively associated with clinical improvement. Finally, Wang et al. [16] analyzed the involvement of serotonin metabolism and reported a significant decrease in urinary 5-hydroxyindoleacetic acid (5-HIAA) levels following FMT. Moreover, specific urinary metabolites—3-hydroxyhippuric acid (3-HHA) and vanillylmandelic acid (VMA)—were associated with core ASD symptoms, including impairments in communication, social interaction, and behavior. Taken together, findings from these studies indicate that FMT induces not only compositional alterations in the gut microbiota but also functional and metabolic shifts, including modulation of neurotransmitters, microbial metabolites, and pathways relevant to the gut–brain axis. The observed donor–recipient engraftment patterns—although partial and variable—may contribute to the clinical response observed in some children with ASD.

Numerous microbiota-targeted interventions have been investigated for their potential to alleviate symptoms of ASD, including probiotics, prebiotics, and synbiotics. Probiotics, particularly those from the *Lactobacillus* and *Bifidobacterium* genera, are among the most commonly studied. A meta-analysis of seven studies evaluating multi-strain probiotic preparations demonstrated a statistically significant improvement in behavioral outcomes, whereas a meta-analysis of four trials investigating single-strain probiotic formulations found no effect on behavioral symptoms in individuals with ASD. Similarly, studies assessing the efficacy of prebiotic and synbiotic interventions have not demonstrated effectiveness in improving behavioral outcomes in individuals with ASD [38]. While multi-strain probiotics may provide short-term improvements in behavioral symptoms, their effects are generally transient and dependent on continuous administration. In contrast, FMT facilitates a more comprehensive and lasting reshaping of the gut microbiota, which may translate into more durable and sustained clinical improvements.

FMT may represent a promising intervention to reduce both GI and autism-related symptoms in children with ASD. However, its efficacy needs to be confirmed in well-designed, placebo-controlled RCTs with adequately powered sample sizes and extended follow-up periods. Outcome assessments should include both immediate post-intervention effects and sustained benefits observed over time. Future studies should also investigate the optimal structure and components of FMT protocols, as the effectiveness of the intervention may depend on multiple procedural variables. Bowel cleansing prior to FMT is likely to improve engraftment and may enhance clinical efficacy, while the role of antibiotic pretreatment remains uncertain and requires further investigation. In addition, it is essential to determine how long the effects of a single FMT dose persist, whether booster doses are necessary, and what the optimal timing between repeated administrations might be. Current evidence suggests that repeated dosing may increase the likelihood of achieving long-lasting effects. The choice of outcome measures should be carefully considered. There is a need to develop and validate assessment tools capable of capturing a broader range of GI symptoms—beyond stool form alone—that are appropriate for children with communication difficulties or their caregivers. Instead of relying on subjective tools such as the GSRS, which evaluates symptom severity and may be imprecise when used by parents, future research could assess the frequency of specific symptoms and their impact on daily functioning, including school attendance, participation in therapeutic activities, and caregiver burden. When assessing ASD-related symptoms, future studies should not rely exclusively on parent-rated scales, which are susceptible to bias, but should also include clinician-administered assessments such as the Autism Diagnostic Observation Schedule (ADOS). ADOS provides a standardized and objective evaluation of core symptoms and may offer a more accurate measurement of treatment effects in clinical trials. Finally, in parallel with clinical outcome assessments, future research should explore the mechanisms underlying FMT efficacy by evaluating multiple dimensions of the gut–brain axis. This includes microbiota profiling, metabolomics, and immune or neurotransmitter-related features. Such integrative approaches may help identify biomarkers predictive of treatment response and guide the development of personalized microbiota-targeted therapies for ASD.

This systematic review has several limitations that should be acknowledged. The small number of high-quality studies, with only two RCTs included, reduces the strength of the evidence. Most data were derived from uncontrolled before-and-after studies with a high risk of bias. Substantial heterogeneity in FMT protocols and outcome measures limits comparability across studies. Moreover, many outcomes relied on parent-reported tools, and follow-up periods were generally short, hindering conclusions about long-term effects.

## 5. Conclusions

FMT shows promise as a safe and potentially effective intervention for alleviating both GI and behavioral symptoms in children with ASD. Emerging evidence suggests that its therapeutic effects may be mediated through modulation of the gut–brain axis, including changes in microbial composition, metabolite profiles, and neuroimmune pathways. Several studies have reported sustained clinical improvements and long-term shifts in gut microbiota. However, current evidence remains insufficient to draw definitive conclusions about its efficacy. Well-designed, placebo-controlled RCTs are needed to confirm these findings and establish standardized protocols for clinical use.

## Figures and Tables

**Table 1:** Characteristics of the included studies.

Author Year Country	Study Design	Sample Size (M/F) Age (Range) yrs	ASD Diagnostic Tool	Presence of GI Symptoms	Intervention	Comparison	GI Symptoms Assessment Tools	ASD-Related Symptoms Assessment Tools	Funding
Kang 2017 [21] USA	Prospective, before-after study	18 (16/2) 7–16	ADI-R	In all children	Standardized human gut microbiota-oral or rectal	None	BSFS, GSRS	CARS, ABC, SRS, VABS-II, PGI-III	Arizona Board of Regents, The Autism Research Institute, Gordon and Betty Moore Foundation grant
N. Li 2021 [23] China	Prospective, before-after study	40 (37/3) 3–17	ADI-R	In all children	Freeze-dried FMT capsules or colonoscopic FMT	None	BSFS, GSRS	ABC, CARS, SRS	Army Medical University, Military Science and Technology Innovation Project, Key Science and Health Joint Project of Chongquing
Zhang 2022 [19] China	Retrospective, before-after study	49 (41/8) 3–14	ADI-R DSM-5	Constipation in 24 children	Washed fecal microbiota (Transendoscopic enteral tubing or nasojejunal tube)	None	BSFS	ABC, CARS	Natural Science Foundation of Guangdong Province, the Guangdong Provincial key disciplines Scientific Research Project of Guangdong Education Department, and the Special Research Project of COVID-19 epidemic Prevention and Control in Colleges and Universities of Guangdong Province
Pan 2022 [18] China	Retrospective, before-after study	42 (34/8) Median 6 (IR 3.75–8.25)	DSM-5	Abnormal fecal form in 21 children	Washed fecal microbiota	None	BSFS	ABC, CARS	Natural Science Foundation of Guangdong Province, Department of Education of Guangdong Province, China Postdoctoral Science Foundation
Liu 2023 [24] China	Prospective, before-after study	24 Sex not reported >2	not stated	not specified	Washed fecal microbiota	None	BSFS	ABC	Innovation Team and Talents Cultivation Program of National Administration of Traditional Chinese Medicine, Guangdong Provincial Key Laboratory of TCM Pathogenesis and Prescriptions of Heart and Spleen Diseases, Special Project for Research and Development in Key areas of Guangdong Province, the Natural Science Foundation of Guangdong Province, Guangdong Key Discipline Research Project of Department of Education of Guangdong Province, COVID-19 Epidemic Prevention and Control Special Research Project of Department of Education of Guangdong Province, Basic and Applied Basic Research Project of Guangzhou Basic Research Program
Chen 2024 [20] China	Prospective, before-after study	29 Sex not reported 2–11	DSM-5	In all children	Freeze-dried FMT capsules	None	BSFS, GSRS	ABC, CARS	Shanghai Hospital Development Center, Clinical Research Plan of SHDC, the Natural Science Foundation of Shanghai, Scientific and Innovative Action Plan of Shanghai, Ministry of Science and Technology of P.R. China, the Special Development Fund of Zhangjiang National Independent Innovation Demonstration Zone, Project of Top Priority Research Center Construct
Y. Li 2024 [22] China	Prospective, before-after study	38 (32/6) 3–14	DSM-5	Present in 31 patients	Lyophilized FMT capsules	None	GSRS	ABC, CARS, SRS	Medical innovation Research project of Shanghai Science and Technology Commission
Wan 2024 [17] China	RCT	113 Sex not reported 4–14	DSM-5	Present in 60 patients	Freeze-dried FMT capsules	Placebo	-	SRS-2, VABS, ABC	National Key Research and Development Program of China, Beijing Natural Science Foundation, General Armament Department, Seventh Medical Center of Chinese PLA General Hospital, Military Family Planning, Foundation of China
Wang 2024 [16] China	RCT	41 (39/3) 4–12	ADOS-2	Not reported	Lyophilized FMT capsules	Placebo	GSRS	CARS, SRS ABC	Natural Science Foundation of Shandong Province

ADI-R—Autism Diagnostic Interview-Revised, DSM-5—5th edition of Diagnostic and Statistical Manual of Mental Disorders, GI—gastrointestinal, ASD—autism spectrum disorder, GSRS—Gastrointestinal Symptom Rating Scale, PGI—Parent Global Impressions, CARS—Childhood Autism Rating Scale, ABC—Aberrant Behavior Checklist, SRS—Social Responsiveness Scale, VABS—Vineland Adaptive Behavior Scale, BSFS—Bristol Stool Form Scale, IR—interquartile range, RCT—randomized controlled trial.

**Table 2:** Model of intervention—FMT working protocols.

Study	Antibiotic Priming	Bowel Cleansing	Stomach Acid Suppressant	Route of Delivery	Dose of Intervention	Duration of Intervention	Follow-Up
Kang 2017 [21]	Oral vancomycin 14 days	1-day liquid diet followed by polyethylene glycol 1 day before FMT	0 meprazole 20 mg/day: from the 5th day before intervention to the last day of intervention	Initial dose: oral FMT mixed in a chocolate milk, milk substitute, or juice or rectal (enema) Maintenance dose: oral	Initial dose: Oral: 2.5 × 10^12^ cells/day for 2 days, divided into 3 daily doses Rectal: single dose 2.5 × 10^12^ cells Maintenance dose: Oral 2.5 × 10^9^ cells/day for 8 weeks (for the oral initial group) or 7 weeks (for the rectal initial group)	10 weeks	18 weeks 2 years
N. Li 2021 [23]	None	Polyethylene glycol 2 L in 2 doses	None	Freeze-dried FMT capsules or colonoscopic FMT	2 × 10^14^ CFU/day	4 one-day sessions, every week	8 weeks
Zhang 2022 [19]	Not reported	Not reported	Not reported	Transendoscopic enteral tubing or nasojejunal tube	120 mL of washed fecal microbiota/day	2 sessions of 6 days, every month	-
Pan 2022 [18]	Not reported	Not reported	Not reported	Transendoscopic enteral tubing	60–90 mL/day, about 5.0 × 10^13^ bacteria/day	At least 2 sessions of 6-days, every month	1 month
Liu 2023 [24]	Not reported	Lactulose 1 day before FMT	Not reported	Transendoscopic enteral tubing	120 mL of washed fecal microbiota/day	4 sessions of 3 days, every month	After FMT session
Chen 2024 [20]	None	None	None	Freeze-dried FMT capsules	Bacterial cells equivalent to 200 g fresh stool/day	4 sessions of 12-days, every month	2 weeks
Y. Li 2024 [22]	None	None	None	Lyophilized FMT capsules	1 g stool/1 kg body weight/session	3 sessions of 3 days every 4 weeks	8 weeks
Wan 2024 [17]	None	None	None	Freeze-dried FMT capsules	80 mg of freeze-dried fecal microbiota/capsule Dose: 8 capsules/day	2 sessions of 6-days, 1st and 5th week of the study	9 weeks 17 weeks
Wang 2024 [16]	None	3 days residue-free semi-liquid diet followed by polyethylene glycol	None	Lyophilized FMT capsules	Not reported	2 sessions of 1-week, 1st and 5th week of the study	9 weeks

**Table 3:** Results of included studies.

Study	GI Symptoms Scales	ASD-Related Symptoms Scales
Kang 2017 [21]	GSRS: Reduction in average score: 82% at week 10, *p* < 0.001, 77% at week 18, *p* < 0.001, 58% after 2 yrs, *p* = 0.01 DSR with a use of BSFS: Reduction in “days with abnormal stools”: 28% at week 10, *p* < 0.002, 30% at week 18, *p* < 0.002, 26% after 2 yrs, *p* < 0.05 Reduction in “hard stools days”: no difference at week 10, 16% at week 18, *p* = 0.002 Reduction in “soft stools days”: 8% at week 10, *p* = 0.03, no difference at week 18 Reduction in “no stool days”: No difference at week 10 and 18	ABC: Reduction in average score: 35% at week 10, *p* < 0.01, 24% after 2 yrs, Improvement and week 18, *p* < 0.01 CARS: Reduction in average score: 23% at week 10, *p* < 0.001, 24% at week 18, *p* < 0.001, 47% after 2 yrs, *p* < 0.001 SRS: Improvement at week 10 and 18, *p* < 0.001 VABS: Increase in average developmental age by 1.4 yrs at week 18, *p* < 0.001, by 2.5 yrs after 2 yrs, *p* < 0.001
N. Li 2021 [23]	GSRS: Reduction in average score: 35% at week 4, <0.0001, Improvement lasted for the next 8 weeks. DSR with a use of BSFS: Reduction in “hard stools days”: 42.5% at week 4, *p* < 0.001, 50% at week 12, *p* < 0.001 Reduction in “soft stools days”: 10% at week 4 and 12, *p* = 0.04	ABC: Improvement at week 4, *p* < 0.0001, Improvement lasted for the next 8 weeks. CARS: Reduction in average score 10% at week 4, *p* < 0.0001, 6% at week 8, *p* < 0.0001 SRS: Improvement at week 4, *p* < 0.0001, Improvement was reversed at week 8 and 12
Zhang 2022 [19]	DSR with a use of BSFS: For constipation group Improvement after 1st and 2nd FMT, *p* = 0.001 For group without constipation No significant improvement after 1st and 2nd FMT	ABC: For constipation group—no difference after 1st FMT, improvement after 2nd FMT, *p* = 0.046, For group without constipation—no difference after 1st and 2nd FMT. CARS: For constipation group—no difference after 1st FMT, improvement after 2nd FMT, *p* = 0.015, For group without constipation—improvement after 1st FMT, *p* = 0.033 and 2nd FMT, *p* = 0.002
Pan 2022 [18]	DSR with a use of BSFS: Reduction in number of children with constipation after 1st to 5th FMT, *p* < 0.05 (No children were constipated after 4th and 5th FMT course) Improvement in number of children with normal fecal form after 2nd to 5th FMT, *p* < 0.05, no difference after 1st FMT	ABC: Improvement after 1st to 5th FMT, *p* < 0.05 CARS: Improvement after 1st to 5th FMT, *p* < 0.05
Liu 2023 [24]	DSR with a use of BSFS: Improvement after 1st to 4th, *p* < 0.05	ABC: Improvement after 1st to 4th, *p* < 0.05
Chen 2024 [20]	GSRS: Improvement at months 1 to 3, *p* < 0.05 DSR with a use of BSFS: Improvement at months 1 to 3, *p* < 0.05	ABC: Improvement at months 1 to 3, *p* < 0.05 CARS: Improvement at month 3, *p* < 0.01, no difference at months 1 and 2
Y. Li 2024 [22]	GSRS: Reduction in average score 51% at week 12, *p* < 0.001, 32% at week 20, *p* < 0.01	ABC: Reduction in average score: 20% at week 12, *p* < 0.0001, 23% at week 20, *p* < 0.001 CARS: Reduction in average score: 10% at week 20, *p* < 0.01 SRS: Improvement at week 12, *p* < 0.001, Reduction in average score: 6% at week 20, *p* < 0.001
Wan 2024 [17]		ABC: No difference between FMT and placebo groups at week 9 and 17 SRS-2: No difference between FMT and placebo groups at week 9 and 17 VABS: No difference between FMT and placebo groups at week 9 and 17 In subgroup of colonization rate ≥20% VABS score increased in FMT group compared to placebo MD 4.93 (95% CI 1.64–8.22)
Wang 2024 [16]	GSRS: Reduction in average score 37% at week 9, *p* < 0.0001 in FMT group, no improvement (*p* = 0.19) in placebo group Significant reduction in FMT group compared to placebo (MD −10.07, 95% CI −13.68–−6.46).	ABC: Improvement in FMT (*p* < 0.0001) and placebo group (*p* = 0.0342) No difference between FMT and placebo groups CARS: Reduction in average score 8% at week 9, *p* < 0.0001 No improvement in placebo group Significant reduction in FMT group compared to placebo (MD −2.12, 95% CI −3.82 to −0.41) SRS: Reduction in average score 9% at week 9, (*p* < 0.0001), no improvement in placebo group Significant reduction in FMT group compared to placebo (MD 12.91 95% CI −21.40–−4.42)

GSRS—Gastrointestinal Symptom Rating Scale, DSR—Daily Stool Record, BSFS—Bristol Stool Form Scale, CARS—Childhood Autism Rating Scale, ABC—Aberrant Behavior Checklist, SRS—Social Responsiveness Scale, VABS—Vineland Adaptive Behavior Scale.

## Supplementary Materials

The following supporting information can be downloaded at https://www.mdpi.com/article/10.3390/nu17132250/s1, Table S1. Search strategy.

## List of Abbreviations

ASD	autism spectrum disorder
FMT	fecal microbiota transplantation
GI	gastrointestinal
ABC	Aberrant Behavior Checklist
CARS	Childhood Autism Rating Scale
SRS	Social Responsiveness Scale
VABS	Vineland Adaptive Behavior Scales
GSRS	Gastrointestinal Symptom Rating Scale
BSFS	Bristol Stool Form Scale
