# Supplementary material for: The Impact of Fecal Microbiota Transplantation on Gastrointestinal and Behavioral Symptoms in Children and Adolescents with Autism Spectrum Disorder: A Systematic Review
Source: Nutrients. 2025 Jul 7;17(13):2250. doi: 10.3390/nu17132250 (PMC12252074; doi:10.3390/nu17132250)
Supplement: Supplementary file 1 [file nutrients-17-02250-s001.zip › nutrients-3707088-supplementary.pdf]

**Supplementary Table S1.** Search strategy

| Database | Search strategy |
| --- | --- |
| Medline via Pubmed | ((autism[Title/Abstract] OR autistic[Title/Abstract] OR asperger[Title/Abstract]) OR ("Autism Spectrum Disorder"[Mesh])) AND ((fecal[Title/Abstract] OR faecal[Title/Abstract] OR microbiota[Title/Abstract] OR microflora[Title/Abstract] OR microbiome[Title/Abstract] OR stool[Title/Abstract] OR feces[Title/Abstract] OR faeces[Title/Abstract] OR gut[Title/Abstract]) AND (transplant*[Title/Abstract] OR transfer[Title/Abstract] OR therap*[Title/Abstract] OR therapy[Title/Abstract] OR transfus*[Title/Abstract] OR implant*[Title/Abstract] OR enema[Title/Abstract] OR treat*[Title/Abstract])) |
| EMBASE | (((fecal OR faecal OR microbiota OR microflora OR bacteria OR microbiome OR stool OR feces OR faeces) NEAR/3 (transplant* OR transfer OR therap* OR transfus* OR implant* OR enema OR treat*)) OR 'fecal microbiota transplantation'/exp) AND (autism OR autistic OR neurodevelopmental OR asperger OR 'autism'/exp) |
| CENTRAL | autism OR autism spectrum disorder OR autistic disorder OR neurodevelopmental disorder OR Asperger syndrome OR neurodevelopmental disorder in Title Abstract Keyword AND fecal microbiota transfer OR fecal microbiota transplantation OR fecal microbiota OR fecal transplant OR fecal transfer OR fecal transplantation OR microbiota transfer OR microbiota transplant OR microbiota transplantation OR fecal |
